# Study on the mechanism of gracillin inhibiting the* proliferation* of lung cancer NCI-H1299 cells based on MAPK signaling pathway

**DOI:** 10.7150/jca.113694

**Published:** 2025-07-01

**Authors:** Bang Xiao, Minhong Zhang, Hai Liu, Aiping Cui, Yihe Tian, Fosheng Tang, Shichen Liao, Mingchun Li, Hao Huang, Weijie Peng, Jianqiong Yang

**Affiliations:** 1 The Clinical Medicine Research Center of the First Clinical Medical College, Gannan Medical University, Ganzhou, China.; 2 School of Rehabilitation Medicine, Gannan Medical University, Ganzhou, China.; 3 Ganzhou Key Laboratory of Antitumor Effects of Natural Products, Gannan Medical University, Ganzhou, China.; 4 Jiangxi Province Key Laboratory of Pharmacology of Traditional Chinese Medicine, Gannan Medical University, Ganzhou, China.; 5 Department of Oncology of the First Clinical Medical College, Gannan Medical University, Ganzhou, China.; 6 Jiangxi Provincal Key Laboratory of Tissue Engineering, Gannan Medical University, Ganzhou, China.; 7 Ganzhou Key Laboratory of Osteoporosis Research, Gannan Medical University, Ganzhou, China.

**Keywords:** Gracillin, Autophagy, MAPK, Non-small cell lung cancer

## Abstract

In this study, we investigated the potential of gracillin, a steroidal saponin compound, as an anticancer agent against non-small cell lung cancer (NSCLC) and explored its impact on autophagy mechanisms. Gracillin significantly inhibited NCI-H1299 cell proliferation and induced autophagic cell death. Mechanistically, gracillin activated the MAPK signaling pathway, evidenced by increased p-ERK and decreased p-JNK levels, suggesting their roles in mediating autophagy induction. Additionally, gracillin upregulated WIPI1 expression, a key autophagy-related protein potentially downstream of the ERK pathway. Evaluation in a xenograft mouse model demonstrated robust anticancer efficacy of gracillin with no significant adverse effects observed. These findings highlight gracillin as a promising candidate for NSCLC therapy, leveraging its ability to induce autophagy through MAPK pathway modulation. Our study provides valuable insights into the therapeutic potential of gracillin and supports its further development as a safe and effective treatment option for NSCLC.

## 1. Introduction

Currently, lung cancer remains the most common type of cancer and the leading cause of cancer deaths in China, accounting for approximately 40% of lung cancer deaths worldwide 1. It is estimated that about 4.82 million people were newly diagnosed with cancer in China in 2022, and about 1.06 million with lung cancer; about 2.57 million people died of cancer, about 730,000 of them died from lung cancer 2. The average 5-year survival rate for lung cancer patients is less than 20% 3, 4. According to the morphological characteristics of lung cancer cells, it can be divided into Non-Small Cell Lung Cancer (NSCLC) and Small Cell Lung Cancer (SCLC), of which NSCLC accounts for 80-85% of lung cancers 5. The treatment of NSCLC broadly includes surgery, radiotherapy, chemotherapy, targeted therapy, immunotherapy 6. Surgical resection is the treatment of choice for early-stage NSCLC. Unfortunately, most patients are already in advanced stages at the time of diagnosis, missing the optimal window for surgery 7, 8. Chemotherapy and radiotherapy, as conventional treatments for NSCLC, can prolong the survival of patients to a certain extent, but the treatment process is prone to drug resistance, and the toxic side effects are severe, seriously affecting the quality of life of patient 9, 10. In recent years, with the continuous development of immunotherapy and targeted therapy, the prognosis of NSCLC patients has improved 11. Targeted drugs against EGFR mutation, ROS1 mutation, ALK mutation may treat some advanced cancers, but the high cost renders them unaffordable for most patients 12. Immune checkpoint inhibitors (ICIs) are novel immunotherapeutic drugs that can enhance the body's own immune system, improving the survival rate of patients with advanced NSCLC 13. Currently, inhibitors targeting programmed cell death-1 (PD-1), programmed cell death ligand-1 (PD-L1), and cytotoxic T-lymphocyte-associated antigen-4 (CTLA-4) have been widely used 14, 15. However, the use of ICIs carries some risks, potentially causing immune-related adverse events in organs such as the skin, gastrointestinal tract, lungs, thyroid, and pituitary gland 16. Therefore, there is an urgent need to identify new, safe, and effective therapeutic targets or agents for lung cancer.

In recent years, the research and application of Traditional Chinese Medicine (TCM) have received widespread attention and achieved remarkable clinical results. Particularly, TCM played an important role in therapeutic practices during the COVID-19 pandemic in China 17. It has been reported that approximately 90% of COVID-19 patients received TCM treatment, with a clinical efficacy rate as high as 80%. The use of TCM treatment also reduced the risk of death in critically ill patients 18. The development and application of antitumor drugs from Chinese herbal medicines have gradually become a hotspot, with natural products serving as important sources of drug discovery. Many natural products extracted from Chinese herbal medicines have shown antitumor activity through various potential mechanisms, demonstrating multi-targeting and low-toxicity properties 19. *Reineckia carnea*, one of the top ten Miao medicines in Guizhou, is often used by Miao people to treat cough and bronchitis 20. Gracillin is a pharmacologically active steroidal saponin compound isolated from *Reineckia carnea*, and its pharmacological effects can be categorized as anti-tumor 21, anti-inflammatory 22, anti-parasitic 23, and anti-pulmonary fibrosis 24, with anti-tumor activity being its main feature. Gracillin has been shown to inhibit the proliferation of human chronic myeloid leukemia K562 cells 25, 26, human cervical cancer Hela cells, human hepatocellular carcinoma H7402 cells 27, human non-small cell lung cancer A549 cells 28, human hepatocellular carcinoma HepG2 cells 29, human leukemia HL60 cells 30, and human melanoma A375 cells 31. All of them have proliferation inhibitory effects. The main pathways of its anti-tumor effects include inhibiting ATP synthesis by affecting mitochondrial function to diminish mitochondria-mediated cellular bioenergetics 21, 32, inducing apoptosis in A549 cells through the mitochondrial pathway 28, inhibiting STAT3 phosphorylation and regulating the expression of STAT3 target gene products 33, and inducing autophagy 31, 34. The above studies indicate that gracillin, as a natural product extracted from the Chinese herb *Reineckia carnea* itself, has a wide range of anticancer effects and multi-targets, and has the potential to be a candidate for antitumor drugs.

Autophagy is the process by which cells degrade and recycle proteins and organelles to maintain intracellular homeostasis. Mammalian autophagy mainly involves the initiation and prolongation of autophagy, formation of autophagosomes, fusion of autophagosomes and lysosomes, and degradation of autophagosome contents. Usually, autophagy plays a protective role in cells, but excessive autophagy can lead to cell death 35, 36. Autophagy plays an important role in tumorigenesis and development and can be involved in the regulation of several intracellular processes such as apoptosis, cell metastasis, iron death, and the cell cycle 36. Targeting autophagy seems to be a promising strategy for tumor suppression 37. In this study, we found that gracillin induced autophagy in NCI-H1299 cells. Transcriptome sequencing revealed that the MAPK signaling pathway might be related to autophagy. Additionally, we found a significant difference in an autophagy-related gene, WIPI1, between the control and drug-treated groups. Human cells have been reported to contain four ATG18 homologs (WIPI1, WIPI2, WIPI3, and WIPI4), with WIPI1 being the first WIPI family member identified to play a role in autophagy 38. During autophagy, WIPI1 localizes to the autophagosome membrane 39. It has been suggested that WIPI1 can mediate autophagosome maturation by specifically binding to PI3P, and the binding of the two is required for the coupling of LC3 to PE 40, 41. In this study, we first verified the inhibitory effect of gracillin on the proliferation of lung cancer cells. We then confirmed the gracillin-induced autophagy phenomenon through various molecular biology experiments. Further experiments clarified the related signaling pathway, and WIPI1, an autophagy-related protein, might be involved in this process. Finally, we verified the anti-tumor effect and *in vivo* safety through *in vivo* experiments.

## 2. Material and Methods

### 2.1. Antibodies and regents

Gracillin was previously extracted from *Reineckia carnea* by our group (HPLC > 98%) 28. 3-methyladenine (3-MA), Cell Counting Kit-8 (CCK-8), Chloroquine (CQ), U0126, SP600125, SB203580, and RIPA Strong Lysate were purchased from Glpbio (Montclair, USA). Fluorescence PCR kit and cDNA Reverse Transcription Kit were purchased from Trans Gen Biotech (Beijing, China). RPMI-1640 and Fetal Bovine Serum (FBS) were purchased from Gibco (Thermo Scientific, USA). Antibodies against SQSTM/P62, Beclin1, and LC3B were purchased from Cell Signaling Technology (Boston, USA). P38, p-P38, GAPDH, HRP-labeled goat anti-rabbit IgG, HRP-labeled goat anti-mouse IgG, CoraLite488-conjugated Goat Anti-Rabbit lgG (H+L) antibody were purchased from Proteintech (Wuhan, China). WIPI1, ERK, p-ERK, JNK, p-JNK antibodies were purchased from Abmart (Shanghai, China). Trizol reagent was purchased from Ambion (Thermo Scientific, USA). The Super ECL Plus sensitizer was purchased from Applygen (Beijing, China). The gene primers used in the experiment were synthesized by Sangon Biotech (Shanghai, China) Co., Ltd.

### 2.2. Cell culture

The NCI-H1299 cell line used in this experiment was purchased from the Shanghai Cell Bank of the Chinese Academy of Sciences. The cells were cultured in RPMI-1640 medium containing 10% FBS and 1% penicillin/streptomycin, and were placed in a cell culture incubator at 37°C with 5% CO_2._

### 2.3. CCK-8 assay for cell viability

NCI-H1299 cells (3 × 10^3^ cells/well) were inoculated into 96-well plates (Corning, USA). After overnight incubation, they were treated with gracillin (0, 0.75, 1.5, 3 μmol/L) for 12, 24, and 48 hours. In the inhibition of autophagy assay viability assay, the NCI-H1299 cells were pre-treated with 3-MA (5 mmol/L), rapamycin (5 μmol/L) for 3 h, and then treated with gracillin (3 μmol/L) for 24 h. After discarding the supernatant, CCK-8 working solution was added to each well and incubated at 37°C for 2 hours. The absorbance at 450 nm was measured using an enzyme marker (VARIOSKAN LUX, ThemoFisher, USA). Cell viability was calculated based on the absorbance value of each group. Cell viability was calculated based on the absorbance values of each group, where cell viability (%) = (average OD value of drug group - average OD value of blank group) / (average OD value of negative control group - average OD value of blank group) × 100%.

### 2.4. Immunofluorescence staining

NCI-H1299 cells (8×10^4^ cells/well) were inoculated into 12-well plates and pretreated with 3-MA (5 mmol/L) for 3h followed by gracillin (3 μmol/L) treatment for 48 hours. After washing with phosphate-buffered saline (PBS) and fixing with 4% paraformaldehyde (Biochem, Shenzhen, China) for 15min, cells were permeabilized by adding 0.5% TritonX-100 solution (Elabscience, Wuhan, China) for 20min. Subsequently, cells were blocked from non-specific antibody interactions by adding a 1% bovine serum albumin (BSA) solution (Elabscience, Wuhan, China) for 1 h. The permeabilized cells were then incubated with the primary antibody (anti-LC3B) at 4°C overnight. Following incubation, cells were washed 3 times with PBS and incubated with the secondary antibody at room temperature for 1 hour. After another 3 washes with PBS, cells were stained with DAPI (Elabscience, Wuhan, China) for 10 minutes at room temperature. Images were acquired using a fluorescence microscope (Axio Vert.A1, ZEISS, GER).

### 2.5. Western blotting

NCI-H1299 cells (2×10^5^ cells/well) were inoculated in 6-well plates. After overnight incubation, cells were incubated with gracillin (0, 0.75, 1.5, 3 μmol/L) for 48 hours. In the experiment using specific inhibitors, CQ (10 μmol/L), U0126 (10 μmol/L), JSP600125 (20 μmol/L), SB203580 (20 μmol/L) were pretreated for 3 h, and then treated with gracillin (3 μmol/L) for 48 h. Cells were then collected and total cellular protein was extracted with RIPA lysis buffer. The protein concentration of each sample was determined by BCA assay. Proteins with different molecular weights were separated by 12% sodium dodecyl sulfate-polyacrylamide gel electrophoresis (SDS-PAGE) and then transferred to polyvinylidene difluoride (PVDF) membranes (Mecrk, GER). Afterwards, the membranes were then blocked with 5% skimmed milk for 1 h. Following blocking, the membranes was cut and incubated with specific primary antibodies overnight at 4°C and then washed three times in TBST buffer. The membrane was incubated with HRP-coupled secondary antibody for 1h at room temperature and then washed 3 times in TBST buffer. Finally, the membranes were developed using ECL reagent, and the gel system captured images and analyzed the gray values of the bands.

### 2.6. Transmission Electron Microscope (TEM)

The cells were inoculated in a 10 cm petri dish and treated with 3μmol/L gracillin for 24h when the cell confluence reached about 70%. Cells were collected by cell scraper and centrifuged in 1.5ml EP tube. Then, pre-cooled 2.5% glutaraldehyde was slowly added along the tube wall and fixed in a refrigerator at 4 °C for 12 h. Then the sample was fixed with 1% osmic acid solution for 1h, and dehydrated with 30%,50%,70%,80%,95%,100% and 100% acetone. The dehydrated samples were embedded and cut into 70 nm thick sections, and stained with uranium acetate and lead citrate. Images were collected under a transmission electron microscope (H-7650, Hitachi, Japan).

### 2.7. Reverse transcription-quantitative polymerase chain reaction (RT-qPCR)

NCI-H1299 cells (2×10^5^ cells/well) were seeded in 6-well plates overnight and co-cultured with gracillin (0, 0.75, 1.5, 3 μmol/L) for 48 h. The cells were collected and total RNA was extracted by Trizol reagent. The cDNA was obtained by reverse transcription and used as a template for target gene amplification. The results were calculated according to the detected Ct value. The number of cycles in which the fluorescence intensity of the cDNA reached the set threshold, with GAPDH as the internal reference gene, ΔCt = the Ct value of the target gene-the Ct value of the internal reference gene, and the relative expression of mRNA in each sample = 2^-ΔΔCt^. The gene primer sequences used in the experiment are as follows:

### 2.8. RNA-sequencing

Control and gracillin (3 μmol/L) groups were set up with four biological replicates in each group, and NCI-H1299 cells were treated with 3 μmol/L gracillin for 24 h. RNA was extracted using Trizol reagent. RNA sequencing was performed by Gene Denovo Biotechnology Co., Ltd (Guangzhou, China) using Illumina HiseqTM 2500/4000. The differential expression threshold was set at FDR < 0.05, |log_2_FC| > 1. The obtained differential genes were analyzed by GO enrichment and KEGG enrichment on the Omicsmart (http://www.omicsmart.com) platform.

### 2.9. Animal experiments

Thirty 4-week-old BALB/c nude mice were purchased from Jiangsu Huachuang Xinnuo Pharmaceutical Technology Co., Ltd., half male and half female, and subcutaneously injected with non-small cell lung cancer cell NCI-H1299 at a concentration of 1 ×10^7^ / 200 μL. When the tumor volume reached 80-90mm^3^, the nude mice were randomly divided into 5 groups, each consisting of 6 mice: the control group (corn oil+5%DMSO), paclitaxel (10 mg/kg), and three gracillin groups (5 mg/kg, 10 mg/kg, and 20 mg/kg). The drug groups were injected six times a week, while the paclitaxel group received injections twice a week for two weeks. The mice were sacrificed by cervical dislocation after anesthesia with excessive inhalation of isoflurane. Nude mice were weighed daily, and tumor dimensions, including long diameter (a) and short diameter (b), were measured using a vernier caliper. The tumor volume calculation formula was V(mm^3^) = a×b^2^×0.5.

### 2.10. Histopathological analysis

Tissues from nude mice (heart, liver, lung, kidney, and tumor) were promptly collected and fixed in 4% paraformaldehyde solution. Following fixation, tissues were embedded in paraffin to prepare 5μm thick sections, which underwent HE staining (heart, liver, lung, kidney) and immunohistochemical staining (tumor).

### 2.11. Determination of liver and kidney function and blood lipid index

Blood samples of each mouse were collected and placed at room temperature for 30 minutes. The supernatant was obtained by centrifugation at 3000 rpm for 15 minutes at 4°C. The related biochemical indexes were detected using an automatic biochemical immune analyzer (Roche, GER), and the data for each index were recorded.

### 2.12. Statistical analysis

All data were presented as mean ± standard deviation (Mean ± SD). Statistical analysis of experimental data was conducted using GraphPad Prism 8.3 software, with differences between multiple groups assessed via one-way analysis of variance (ANOVA). Each experiment was replicated at least three times, with statistical significance defined as *P* < 0.05.

## 3. Results

### 3.1. Gracillin inhibits the proliferation of H1299 cells

**Figure 1:**
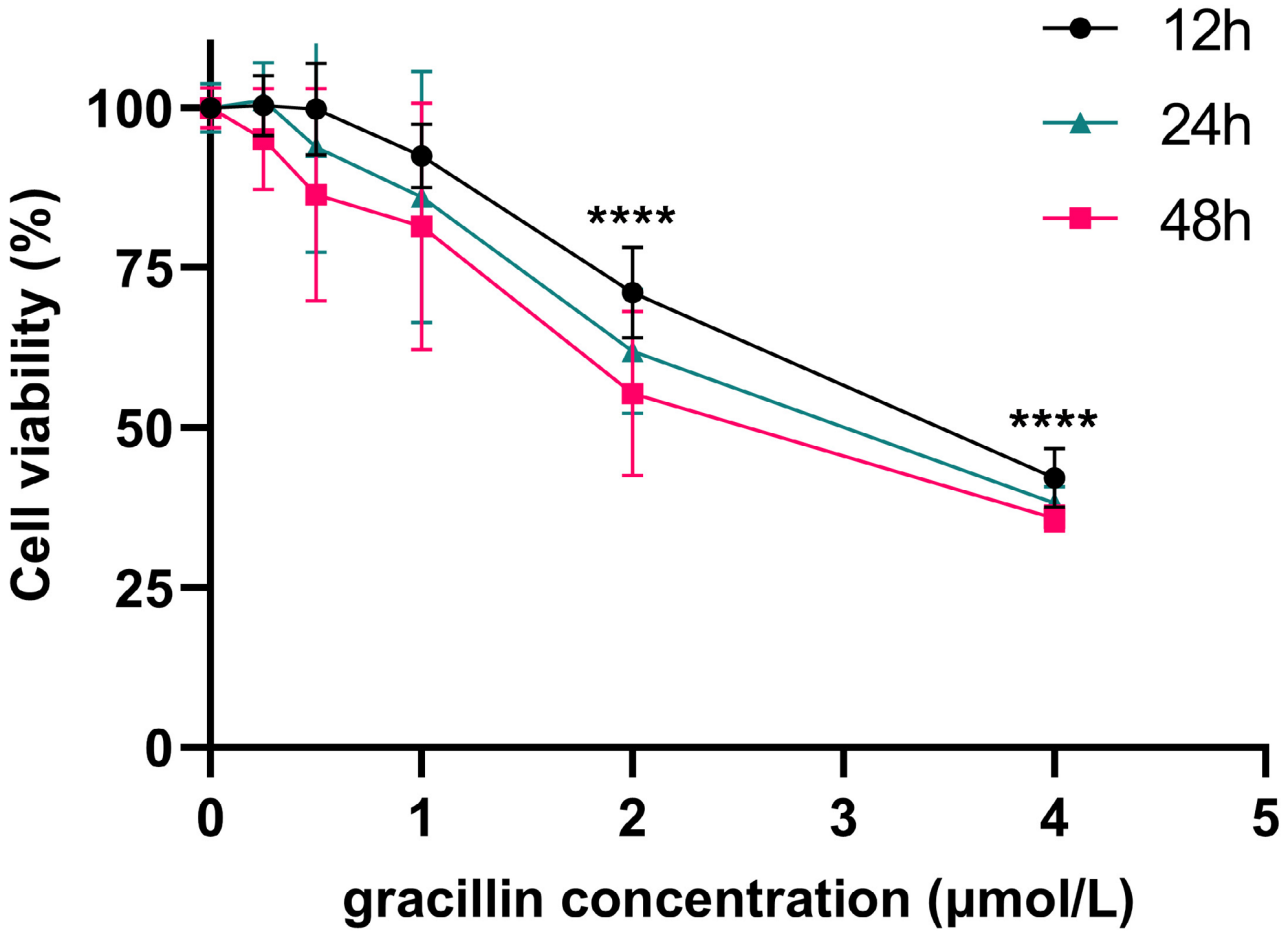
Effect of gracillin on the proliferation of NCI-H1299 cells.

CCK-8 is a rapid and highly sensitive detection reagent based on WST-8(2-(2-methoxy-4-nitrophenyl)-3-(4-nitrophenyl)-5-(2,4-disulfophenyl)-2H-tetrazole monosodium salt) for cell proliferation and cytotoxicity. Its working principle is that in the presence of electron coupling reagents, WST-8 can be reduced by mitochondrial dehydrogenase to orange-yellow formazan products, and its color depth is proportional to the degree of cell proliferation 42. To investigate the impact of gracillin on the viability of human non-small cell lung cancer cells, we treated H1299 cells with various concentrations of gracillin (0, 0.25, 0.5, 1, 2, 4 μmol/L) for 12, 24, and 48 hours. The viability of H1299 cells was determined by CCK-8 method. Compared to the control group, gracillin significantly decreased the viability of H1299 cells, with the degree of proliferation inhibition influenced by both concentration and duration (Fig. 1). The IC_50_ value of gracillin on H1299 cells after 24 hours was determined to be 2.84 μmol/L. Based on this, we selected 3 μmol/L as the highest experimental concentration of gracillin in the subsequent experiments.

### 3.2. Gracillin affects the changes of autophagy-related indexes in H1299 cells

**Figure 2:**
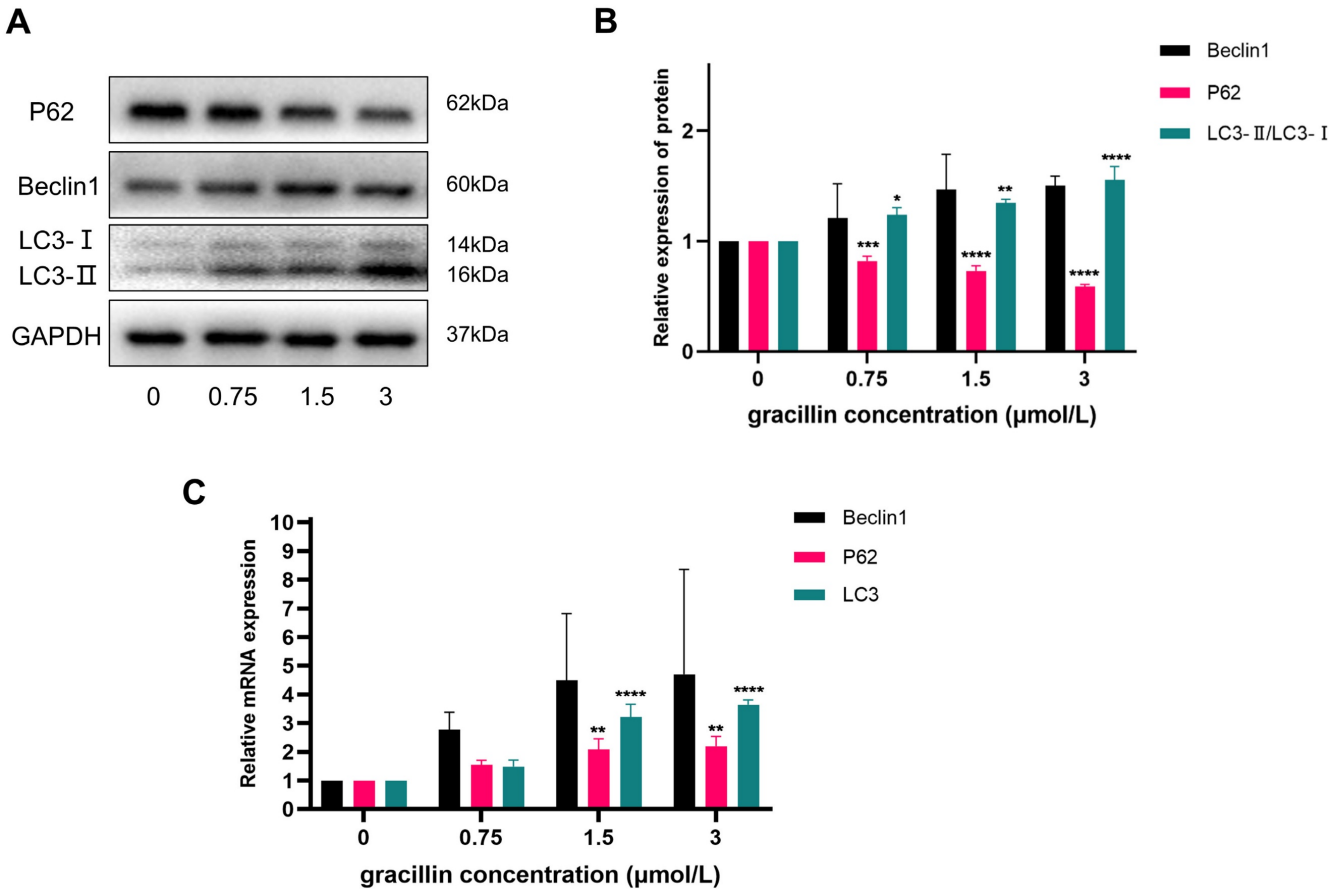
Effects of gracillin on autophagy-related indexes. NCI-H1299 cells were treated with different concentrations of gracillin for 48 h. NCI-H1299 cells were treated with different concentrations of gracillin for 48 h. (A) P62, Beclin1, LC3 protein band map;(B) Compared with the control group, the results of semi-quantitative analysis of P62, Beclin1 and LC3 proteins;(C) Expression analysis of autophagy-related genes Beclin1, P62, and LC3. Compared with the control group, *P < 0.05, **P < 0.01, ***P < 0.001, ****P < 0.0001.

In order to investigate the effect of gracillin on autophagy-related indexes in H1299 cells, this experiment treated H1299 cells with different concentrations of gracillin for 48h. The expression of autophagy-related proteins and genes was then detected using RT-qPCR and Western blot. The experimental results showed that the mRNA levels of autophagy-related genes Beclin1, LC3, and P62 were up-regulated to varying degrees in the gracillin-treated cell group compared with the untreated control group (Fig. 2). At the protein level, the expression of Beclin1 showed a tendency to be up-regulated with increasing drug concentration, whereas the protein level of P62 was subsequently down-regulated, a phenomenon that was also concentration-dependent (Fig. 2A, B). Of particular importance, the ratio of LC3-II to LC3-I was significantly elevated in the drug-treated group, a change that is a key marker for the activation of the autophagic process. In conclusion, gracillin was able to influence the changes in the expression of autophagy proteins in H1299 cells.

### 3.3. Gracillin induces autophagy in H1299 cells

**Figure 3:**
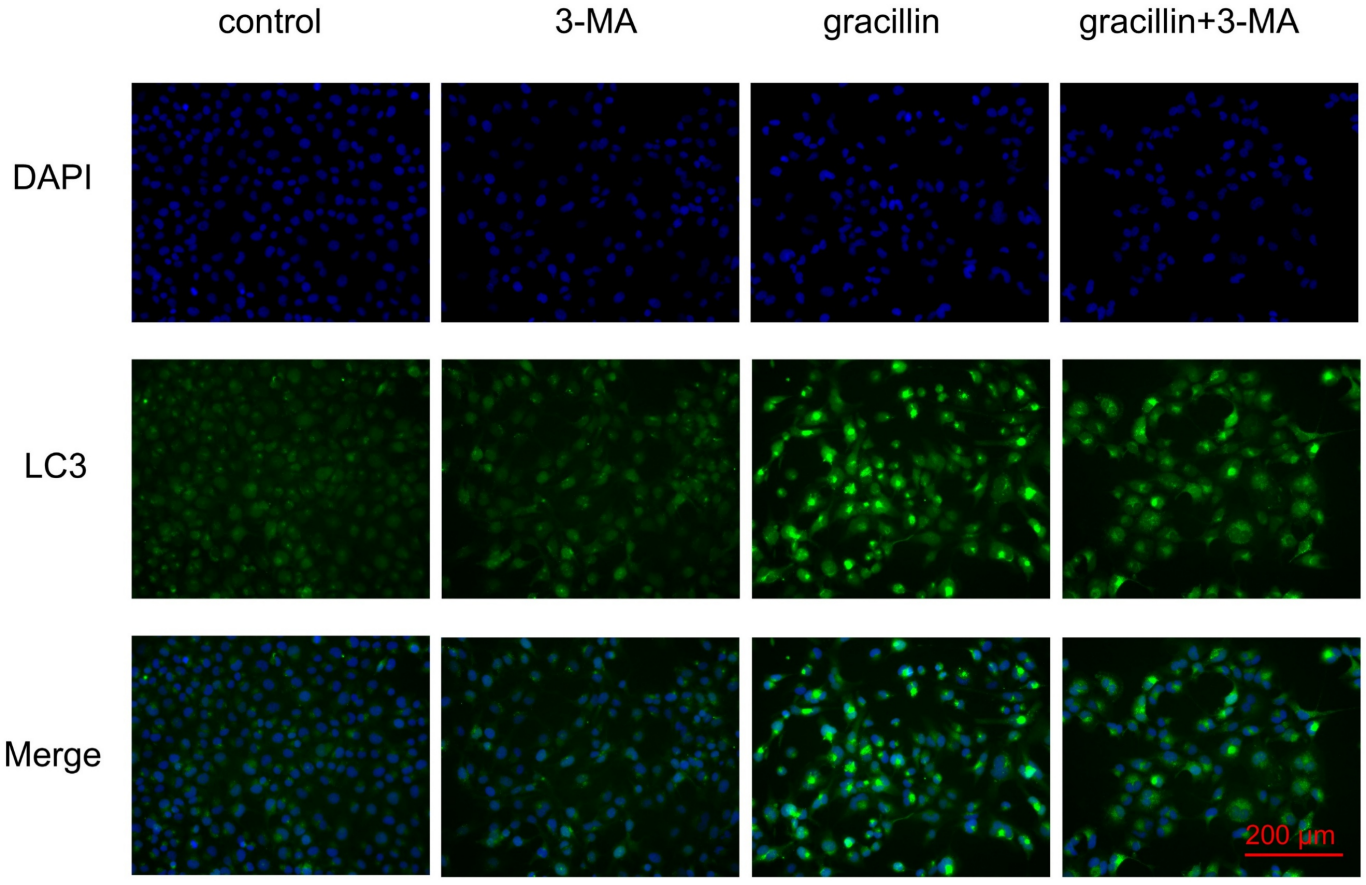
Immunofluorescent co-labeling of LC3 (green) and nuclei (blue).

**Figure 4:**
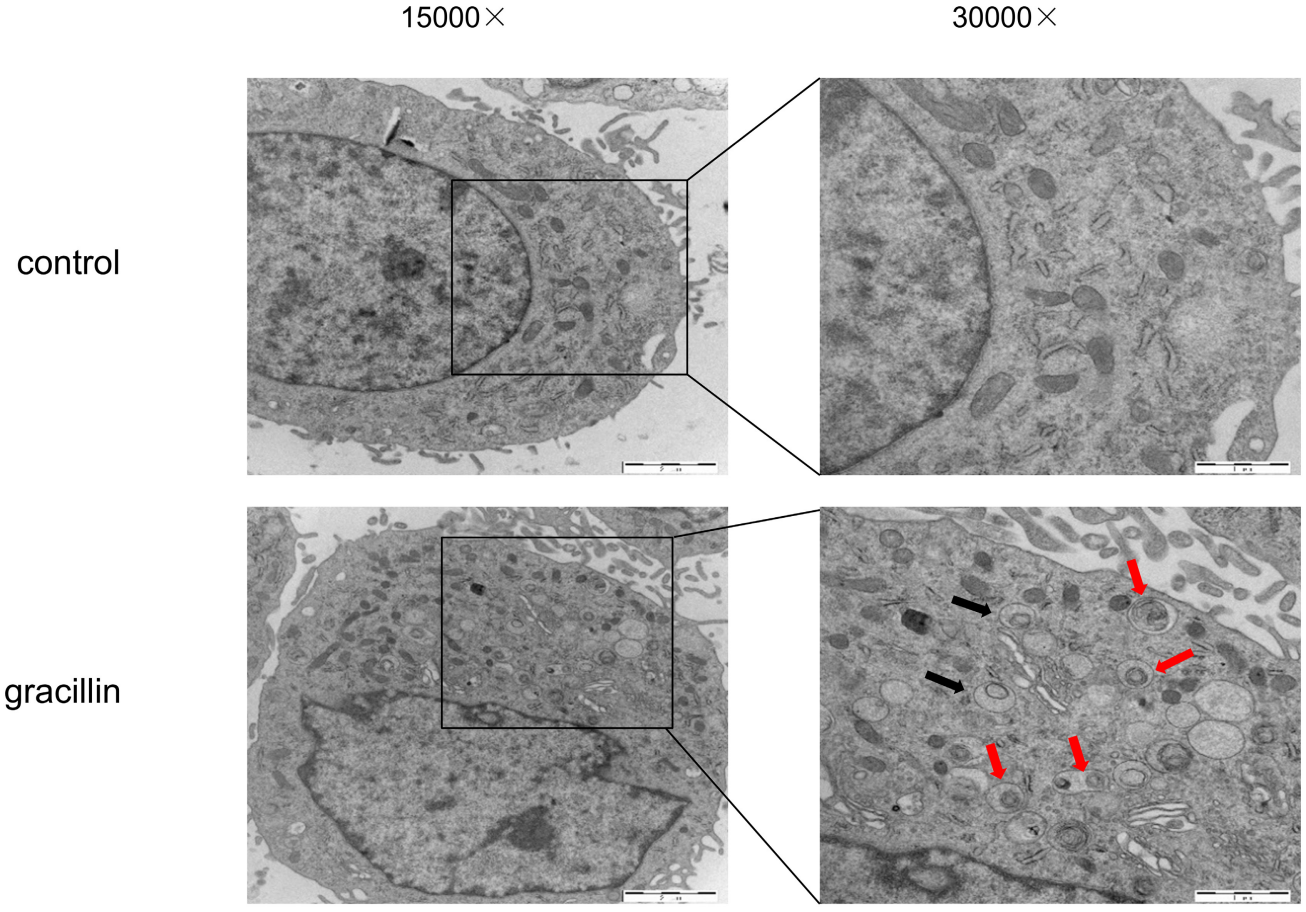
H1299 cells were treated with or without gracillin (3 μmol/L), and autophagosomes (black arrow) or autolysosomes (red arrows) were observed using TEM.

Under normal conditions, cellular autophagy levels are low, and LC3 exists in the cytoplasm in its unmodified form, LC3-I. When cells receive autophagic signals, the LC3-II protein, which undergoes modification, binds to the membrane of autophagic vesicles to form green dot-like structures, playing a key role in the formation and expansion of autophagic vesicles. Therefore, autophagy can be better detected by immunofluorescence with LC3. We found that treatment of H1299 cells with 3 μmol/L gracillin alone induced distinct fluorescent dots of LC3 aggregates (Fig. 3). In contrast, in control H1299 cells, the structure of the LC3 fluorescent dots was essentially absent. 3-MA is an autophagy inhibitor that inhibits the formation of autophagosomes. The group of cells co-treated with 3-MA (5 mmol/L) and gracillin showed significantly fewer LC3 fluorescent dots compared to the group treated with gracillin alone. These results strongly indicate that gracillin upregulates autophagy levels in H1299 cells. Transmission electron microscopy images directly indicate the formation of autophagosomes, characterized by a vacuolar structure of double or multilayer films under the microscope. After treatment with gracillin (3 μmol/L), a large number of autophagic vacuoles were observed in the cells (Fig. 4). Therefore, this study demonstrates that gracillin induces autophagy in human lung cancer cells.

### 3.4. Gracillin can induce autophagic flux in H1299 cells

**Figure 5:**
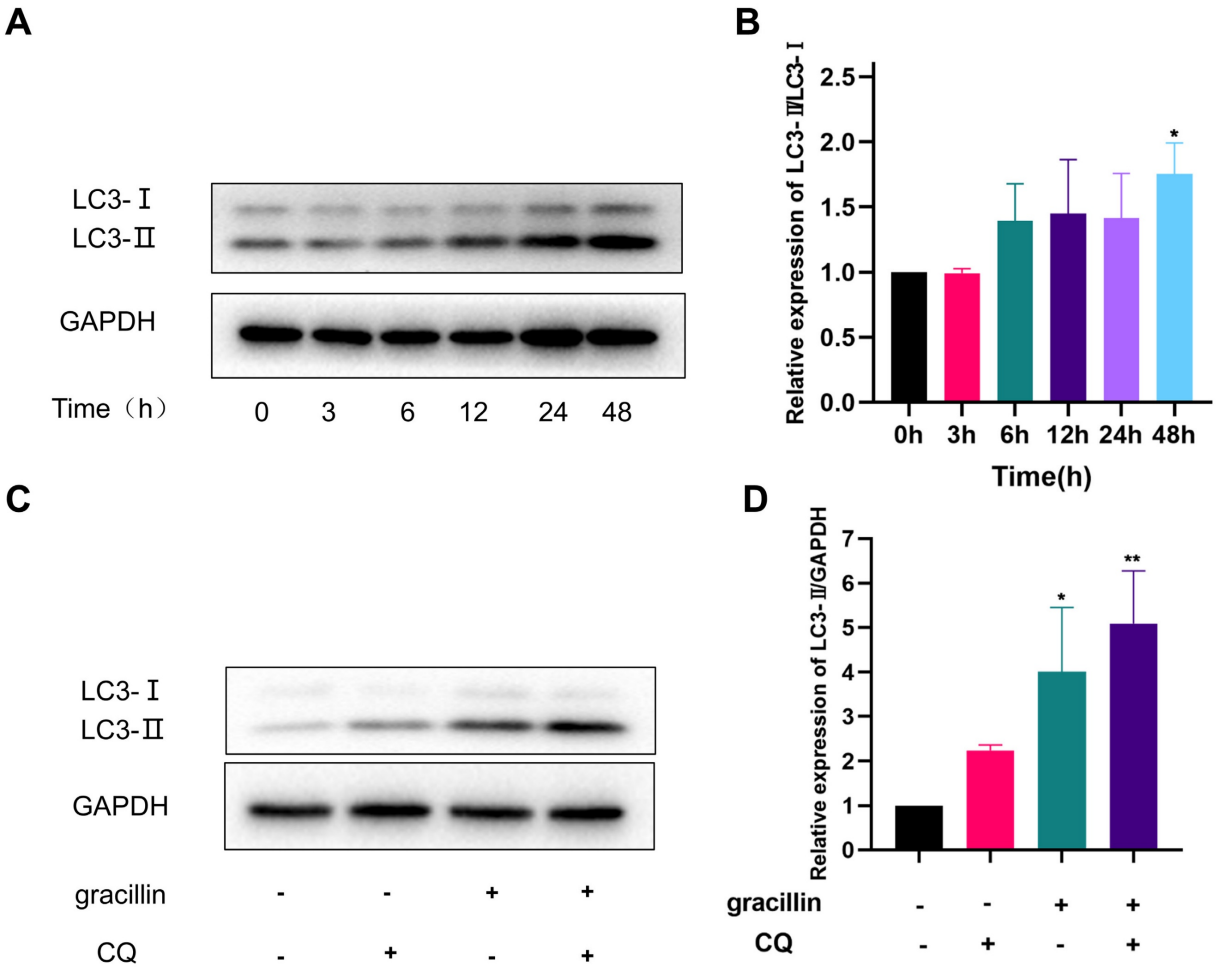
Gracillin induces autophagic flow. (A) LC3 protein banding plots of gracillin-treated NCI-H1299 at different times; (B) Semi-quantitative analysis of LC3 protein compared with control; (C) Banding plots of autophagic flow verified by chloroquine intervention; (D) Semi-quantitative analysis of LC3-II in autophagic flow verified by chloroquine intervention. Compared with the control group, *P < 0.05, **P < 0.01.

Firstly, the LC3-II protein of H1299 cells treated with 3 μmol/L gracillin for different times (0,3,6,12,24,48h) was quantitatively analyzed by Western blot. The results of the Western blot showed that the expression level of LC3-II protein increased in the early stage (6-12 h) and continued to increase with prolonged gracillin treatment time, reaching its peak at 48 h (Fig. 5A, B). Generally speaking, there are two reasons for the increase in LC3-II protein expression in cells. One is that the level of autophagy is enhanced, leading to a conversion of a large number of LC3-I types in cells to LC3-II types. Another reason is that the normal autophagy pathway is blocked, preventing the fusion of lysosomes and autophagosomes 43. To investigate the reason for gracillin-induced increase in LC3-II in H1299 cells, we used the small molecule drug CQ and found that when H1299 cells were co-treated with gracillin (3 μmol/L) and CQ(10 μmol/L) for 48 hours, the increase in LC3-II type was significantly higher than that in either the gracillin group or the CQ group alone, indicating that gracillin can indeed induce autophagic flux (Fig. 5C, D).

### 3.5. Autophagy inhibitors partially reversed the inhibitory effect of gracillin on proliferation

**Figure 6:**
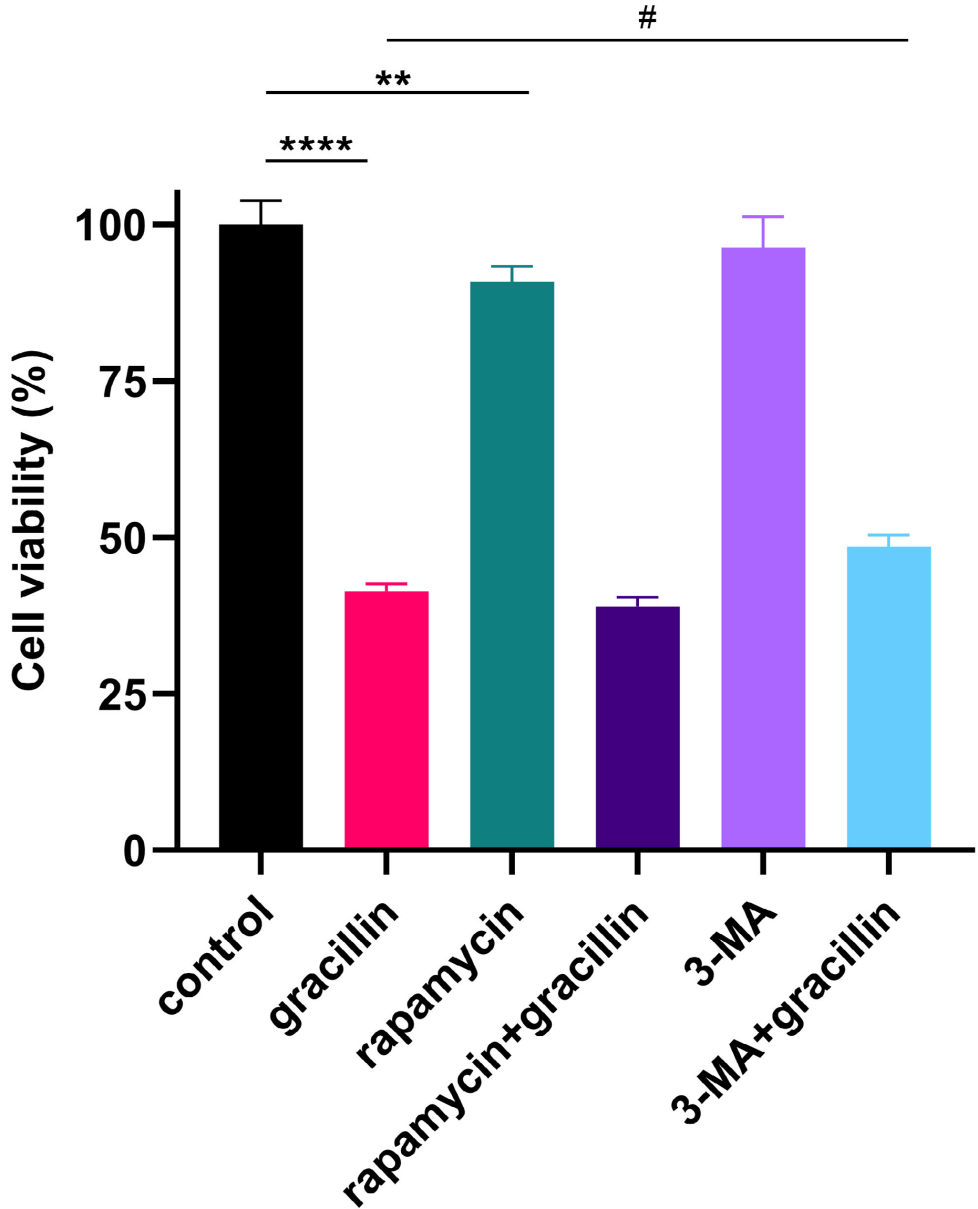
Effects of inhibiting or promoting autophagy on cell viability. Compared with the control group, **P < 0.01, ****P < 0.0001; gracillin group compared with 3-MA+gracillin group, #*P* < 0.05.

To further validate the role of gracillin-induced autophagy in lung cancer cells, cells were treated with 3 μmol/L gracillin in the presence or absence of the early autophagy inhibitor, 3-MA (5 mmol/L), or the autophagy activator, rapamycin (5 μmol/L). The results are shown in Figure 6, where cell viability was significantly reduced by gracillin compared with control, and cell viability was significantly increased after treatment with gracillin in the presence of 3-MA. In summary, gracillin may inhibit the proliferation of NCI-H1299 cells partly by promoting autophagy.

### 3.6. Transcriptome sequencing analysis of NCI-H1299 cells

**Figure 7:**
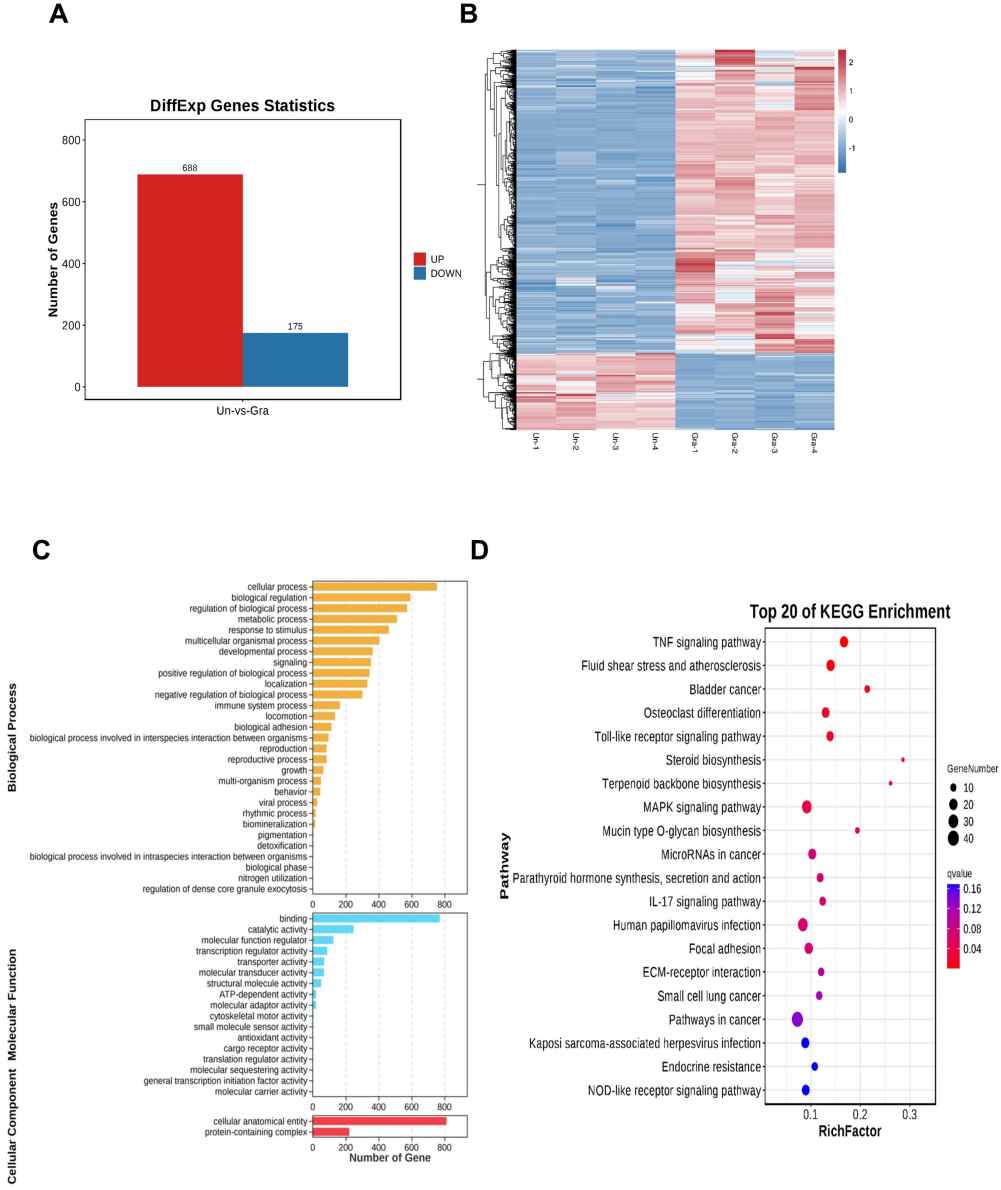
(A) Differential gene histogram; (B) Clustering heatmap differential gene enrichment results (C) GO functional enrichment analysis; (D) KEGG functional enrichment analysis.

After treating NCI-H1299 cells with 3 μmol/L gracillin, total cellular RNA was collected for transcriptome sequencing. The screening conditions were defined as |log_2_FC| > 1.0 and FDR < 0.05, resulting in a total of 863 differentially expressed genes, including 688 significantly upregulated genes and 175 significantly downregulated genes (Fig. 7A). To visualize the differences between the treatment and control groups, the 863 differentially expressed genes were clustered and analyzed, and the clustering heatmap is shown in Figure 7B. To further elucidate the potential mechanism of gracillin-induced autophagy in NCI-H1299 cells, we performed GO and KEGG functional enrichment analysis of the differentially expressed genes identified by transcriptome sequencing, aiming to explore the pathways in which these genes were enriched (Fig. 7C, D). Figure 7D displays the KEGG enrichment results: the horizontal axis represents the number of differential genes annotated to the KEGG pathway, while the vertical axis represents the KEGG pathway itself. The size of the dots corresponds to the number of genes annotated to the pathway, and the color gradient from red to purple indicates the significance of enrichment, with redder colors indicating higher significance. Figure 7B illustrates the top 20 pathways enriched by differential genes. The TNF signaling pathway, Toll-like receptor signaling pathway, and MAPK signaling pathway might be the main pathways through which gracillin exerts its proliferation-inhibiting effects. Some studies have indicated the involvement of the MAPK pathway in autophagy. Notably, the enrichment results for the MAPK signaling pathway were highly significant, with the highest number of differential genes annotated to this pathway. Considering these findings, we primarily focused on the MAPK signaling pathway in this study.

### 3.7. Effects of Gracillin on the MAPK Pathway

**Figure 8:**
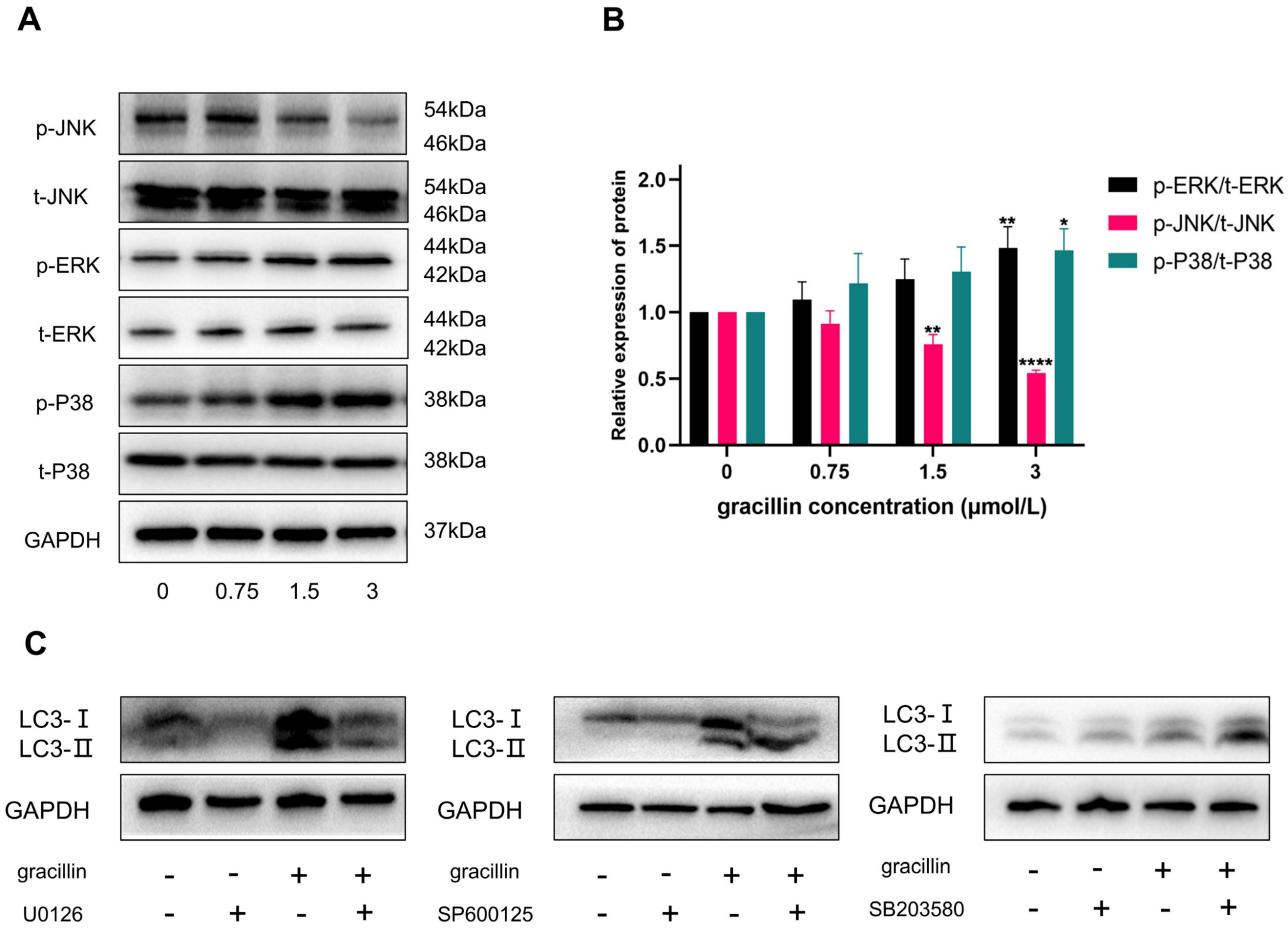
Effect of Gracillin on MAPK pathway. (A) MAPK signaling pathway protein bar graph; (B) Semi-quantitative analysis of MAPK signaling pathway proteins compared with the control group; (C) Effect of inhibiting MPAK pathway on LC3-II. Compared with the control group, *P < 0.05, **P < 0.01, ****P < 0.0001.

The MAPK signaling pathway plays a crucial role in various physiological and pathological processes in eukaryotic cells, including proliferation, differentiation, apoptosis, autophagy, and cell cycle arrest. Based on the results of KEGG pathway enrichment analysis, to determine whether gracillin could regulate the MAPK signaling pathway, we assessed the regulatory effects of gracillin on key proteins in the MAPK pathway using Western blot analysis. The results are shown in Figure 8A, B. After 48 hours of treatment with different concentrations of gracillin, no significant changes were observed in t-ERK, t-P38, or t-JNK, while p-ERK and p-P38 were significantly upregulated, and p-JNK was significantly downregulated compared with the control group.

To further investigate whether gracillin-induced autophagy was dependent on p-ERK, p-JNK, and p-P38, cells were pretreated with 10 μmol/L U0126 (ERK inhibitor), 20 μmol/L SP600125 (JNK inhibitor), and 20 μmol/L SB203580 (P38 inhibitor) for 3 hours, followed by treatment with or without 3 μmol/L gracillin for 48 hours. As shown in Figure 8C, pretreatment with U0126 (ERK inhibitor) significantly reduced LC3-II accumulation in NCI-H1299 cells, and further inhibition of JNK also promoted LC3-II accumulation. In contrast, SB203580 (P38 inhibitor) pretreatment increased the accumulation of LC3-II in the cells, suggesting that the activation of P38 might not be involved in activating autophagy. In summary, gracillin may induce autophagy in NCI-H1299 cells by activating the ERK pathway and inhibiting the JNK pathway.

### 3.8. Autophagy protein WIPI1 is highly expressed at the gene and protein level

**Figure 9:**
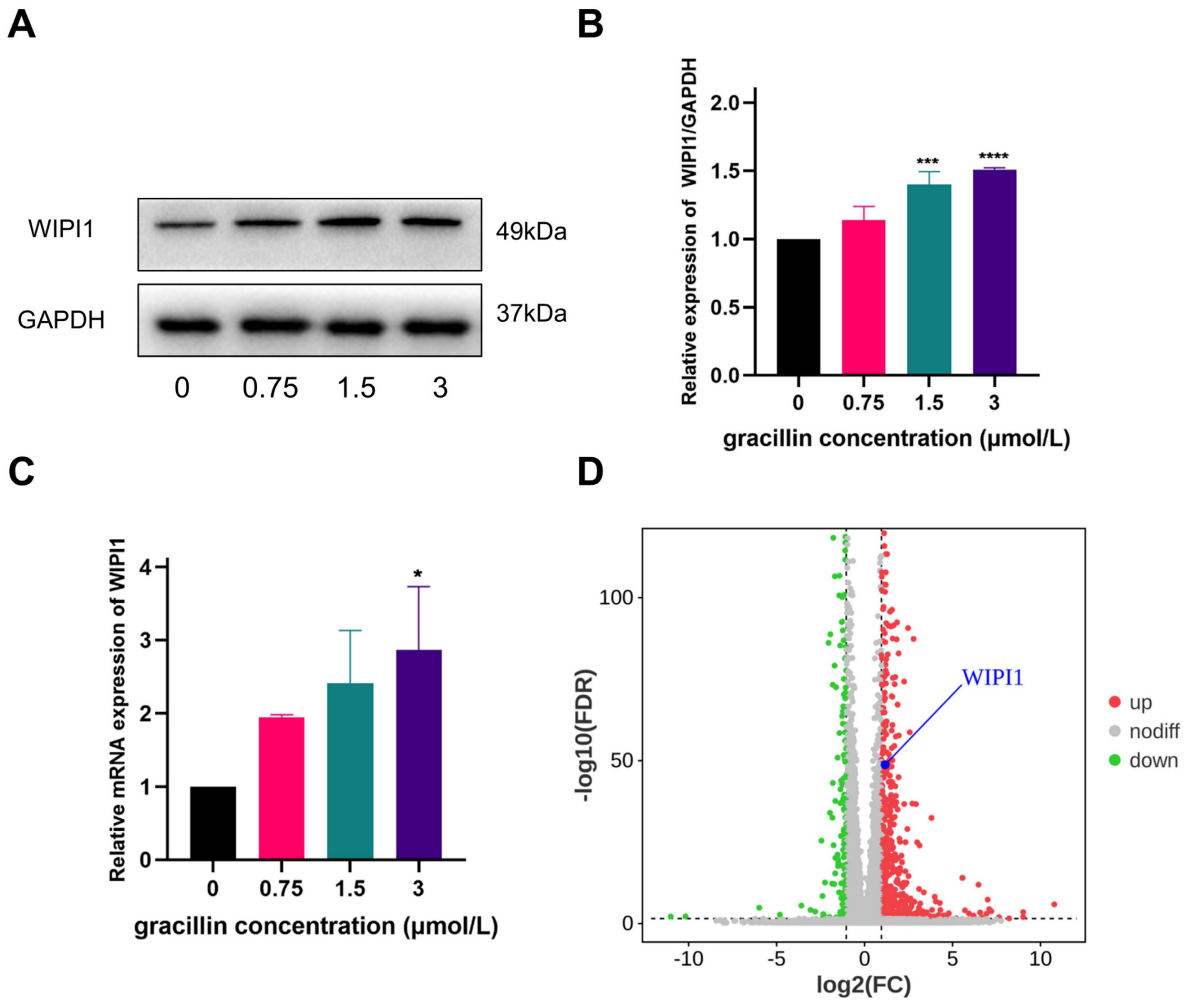
Effect of gracillin on WIPI1. (A) WIPI1 protein bar graph; (B) Semi-quantitative results of WIPI1 protein compared to control; (C) Effect of gracillin on WIPI1 mRNA; (D) Display of differentially expressed gene WIPI1 in volcano plot. Compared with the control group, *P < 0.05, ***P < 0.001, ****P < 0.0001.

**Figure 10:**
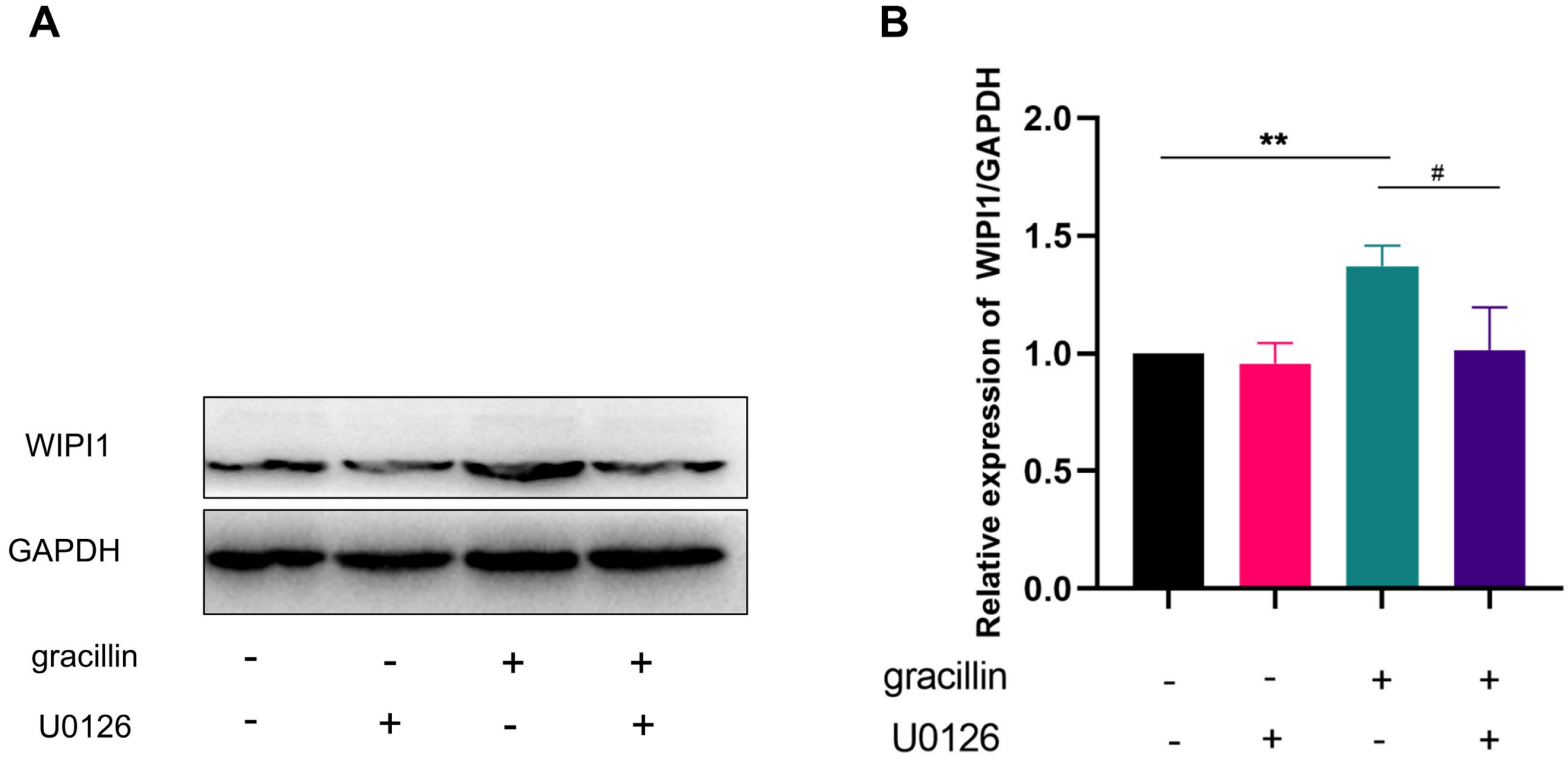
effects of inhibiting ERK pathway on WIPI1 protein. (A) Protein bar graph of WIPI1 after inhibition of ERK pathway; (B) Semi-quantitative results of WIPI1 protein compared with control group. Compared with the control group, **P < 0.01; gracillin group compared with U0126+gracillin* group*, ^#^*P* < 0.05.

After treating cells with different concentrations of gracillin (0,0.75,1.5,3 μmol/L), we verified changes in WIPI1 expression through RT-qPCR and Western blot experiments. The results, as shown in Figure 9A-C, indicate a significant upregulation of WIPI1 at both the protein and gene levels following gracillin treatment, consistent with findings from transcriptome sequencing (Fig. 9D). When cells were pretreated with the ERK pathway inhibitor U0126, WIPI1 protein expression was inhibited, suggesting that inhibition of the ERK pathway affects the upregulation of WIPI1 protein induced by gracillin, as depicted in Figure 10 A, B. In summary, gracillin upregulates WIPI1 expression, and WIPI1 may function as a downstream factor of the ERK pathway.

### 3.9. Gracillin inhibits the growth of transplanted tumors *in vivo*

**Figure 11:**
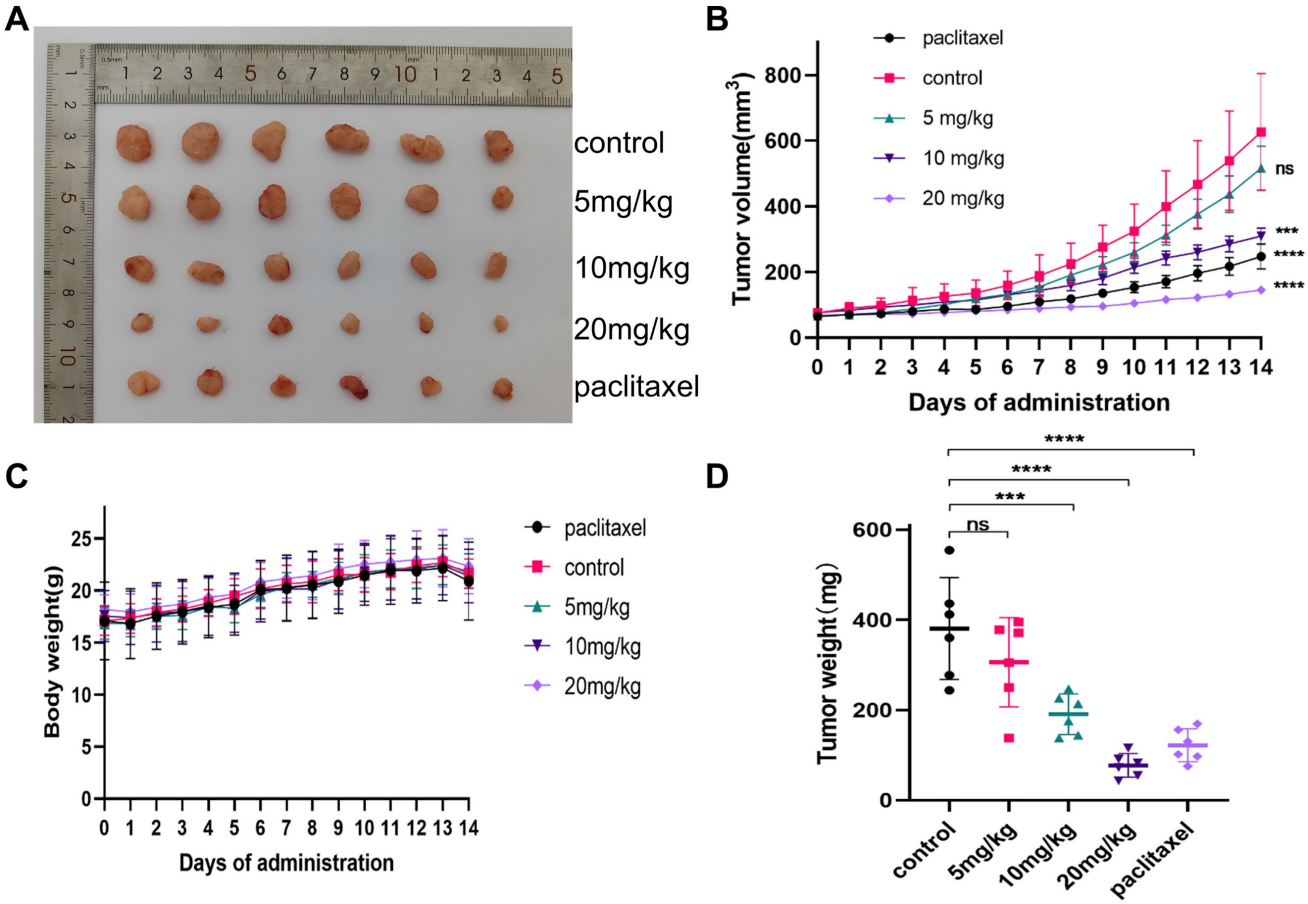
gracillin inhibits the growth of NCI-H1299 cell xenograft tumor. (A) Tumors after different doses of gracillin treatment; (B) Effect of different doses of gracillin treatment on tumor volume; (C) Effect of different doses of gracillin treatment on body weight of nude mice; (D) Tumor weight. Compared with the control group, *P < 0.05, ***P < 0.001, ****P < 0.0001.

To verify whether gracillin could inhibit the proliferation of NCI-H1299 cells *in vivo*, the anticancer activity of gracillin was evaluated by establishing a xenograft tumor model. NCI-H1299 hormonal nude mice were treated with different doses of gracillin, and no deaths were observed among the nude mice during the experimental period. As illustrated in Figure 11B, the transplanted tumor volume increased over time, but the tumor volumes in the gracillin-treated group were consistently smaller than those in the control group (Fig. 11A). The inhibitory effect of gracillin on the weight and volume of NCI-H1299 transplanted tumors showed a dose-dependent relationship compared to the control group (Fig. 11C, D), with the average tumor weights of the 5 mg/kg, 10 mg/kg, and 20 mg/kg groups reduced by 19.54%, 37.71%, and 59.50%, respectively.

**Figure 12:**
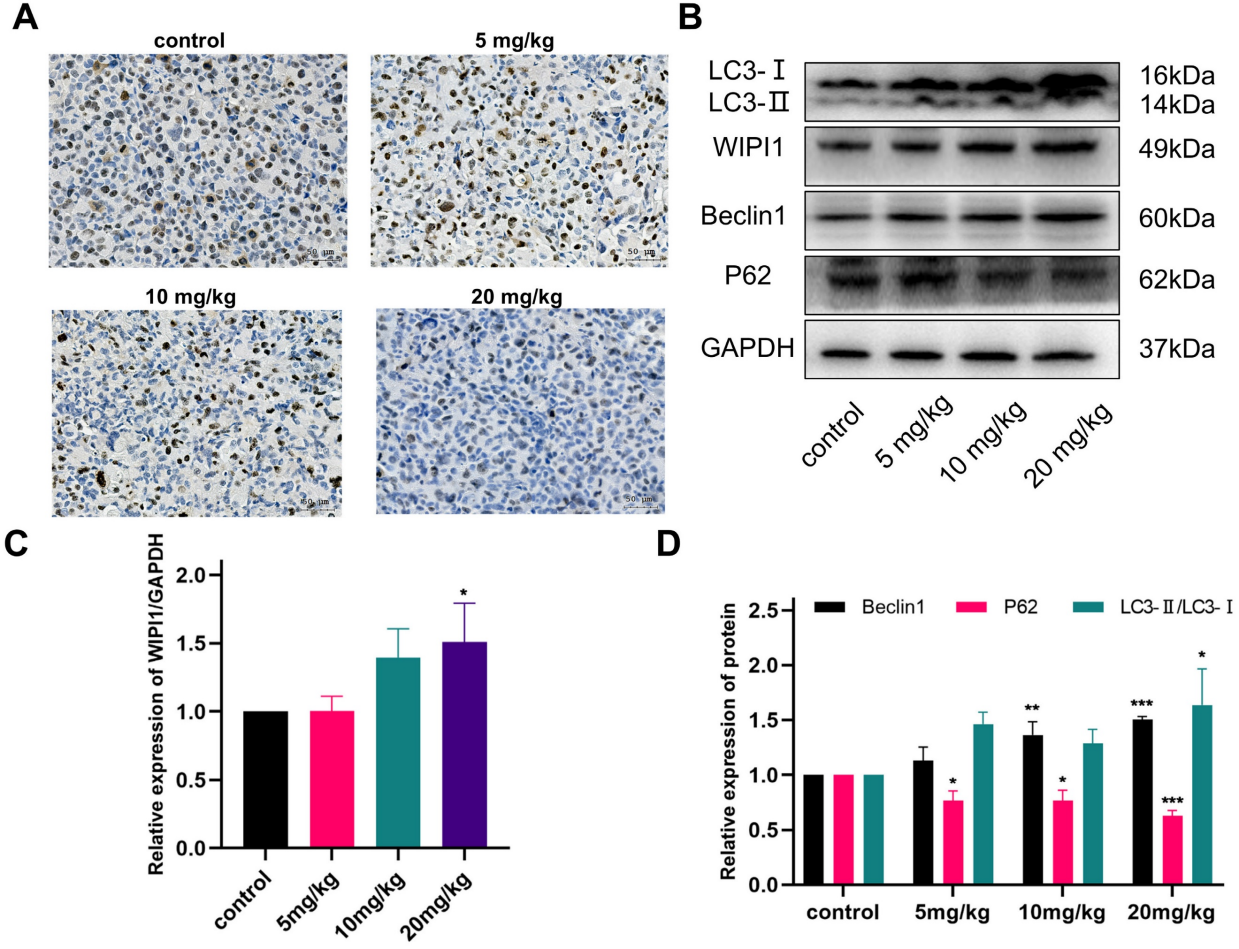
Effect of gracillin on autophagy protein expression in tumor tissues *in vivo*. (A) Ki-67 immunohistochemical staining of tumor tissue sections, scale bar = 50 μm; (B) Autophagy proteins LC3, Beclin1, WIPI1, and P62 banding diagrams; (C) Semi-quantitative results of WIPI1 proteins compared with the control group; (D) Semi-quantitative results of Beclin1, P62, and LC3 proteins. Compared with the control group. *P < 0.05, **P < 0.01, ***P < 0.001.

Ki-67 is currently the most widely used marker of cancer cell proliferation 44. It is a protein present in the nucleus of cells and is expressed in all cell cycle phases—G1, S, G2, and M phases—being highest at the beginning of the M phase, and is not expressed in the resting phase, G0 phase. Therefore, its expression level represents the degree of active cell proliferation 45. To further verify the effect of gracillin on the proliferation of NCI-H1299 cells, the expression of Ki-67 in tumor tissues was detected by immunohistochemistry. The results in Figure 12A showed that Ki-67 protein expression in the gracillin-treated group was lower than that in the control group, indicating its inhibitory effect on the proliferation of NCI-H1299 cells *in vivo*.

As shown in the results of Figure 12B-D, the autophagy-related proteins LC3, Beclin1, and WIPI1 were upregulated to varying degrees in tumor tissues after gracillin treatment, and the expression of P62 was significantly reduced, consistent with the *in vitro* findings. This result indicates that gracillin can inhibit the growth of NCI-H1299 tumors in nude mice subcutaneously by promoting autophagy.

### 3.10. Evaluation of *in vivo* safety

**Figure 13:**
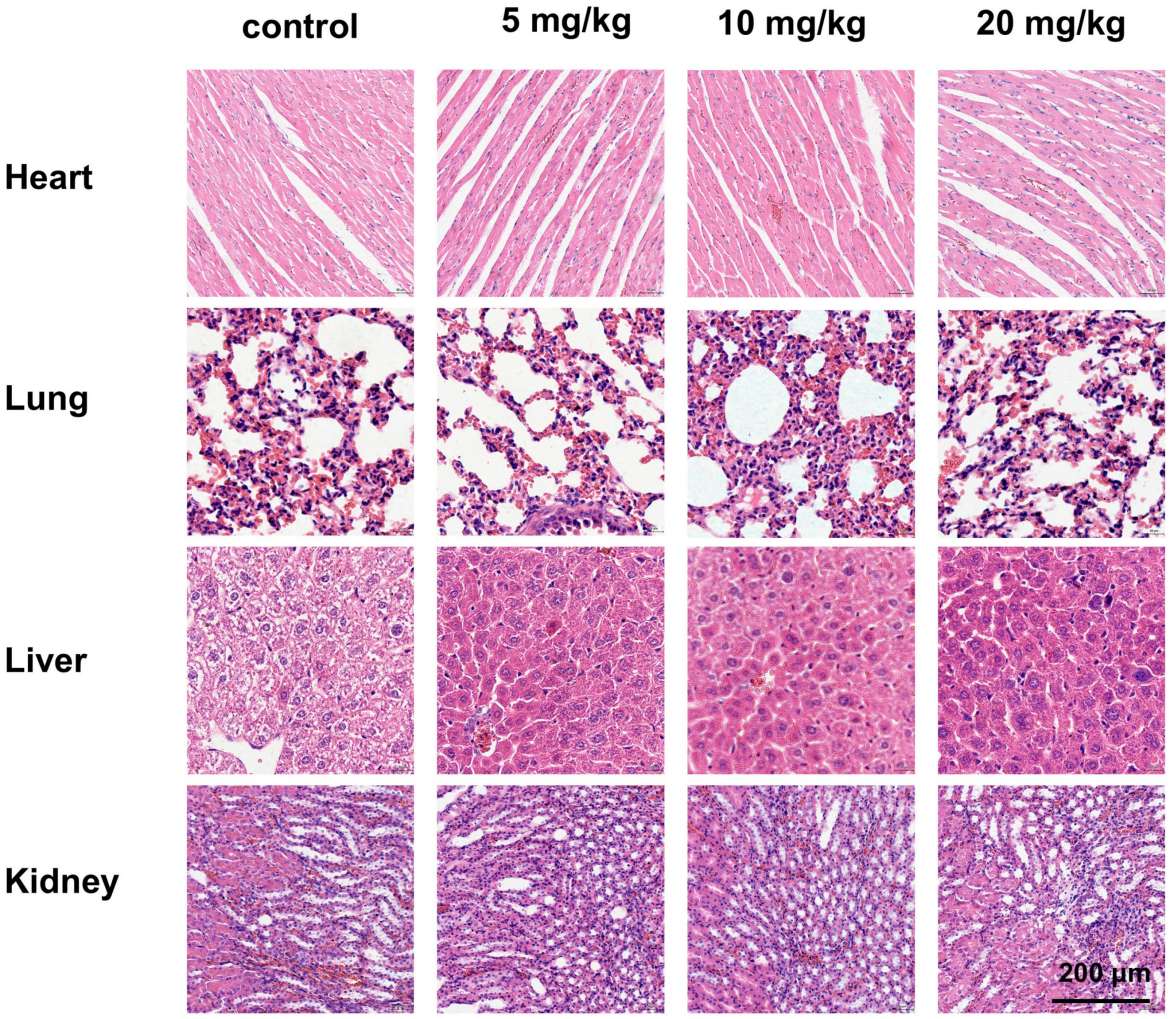
HE staining of heart, liver, lungs and kidneys after different doses of treatment, scale bar = 200 μm.

In addition to evaluating the antitumor effects of gracillin, we also examined its safety in nude mice. Throughout the administration period, we observed no significant difference in the body weight of tumor-bearing nude mice in the drug-treated group compared to that of the control group (Fig. 11C), indicating that gracillin had no obvious systemic toxicity. Hematoxylin and eosin (HE) staining revealed normal morphology in various organs and tissues. Myocardial tissue cells appeared neatly aligned and structurally intact, showing no signs of atrophy, necrosis, degeneration, or hypertrophy. Lung tissue exhibited a clear structure, with no thickening, congestion, or edema in the alveoli. Liver cells were neatly arranged, with abundant cytoplasm and large, round nuclei. Glomeruli and renal capsules were clearly visible, with no evidence of swelling or necrosis (Fig. 13).

**Figure 14:**
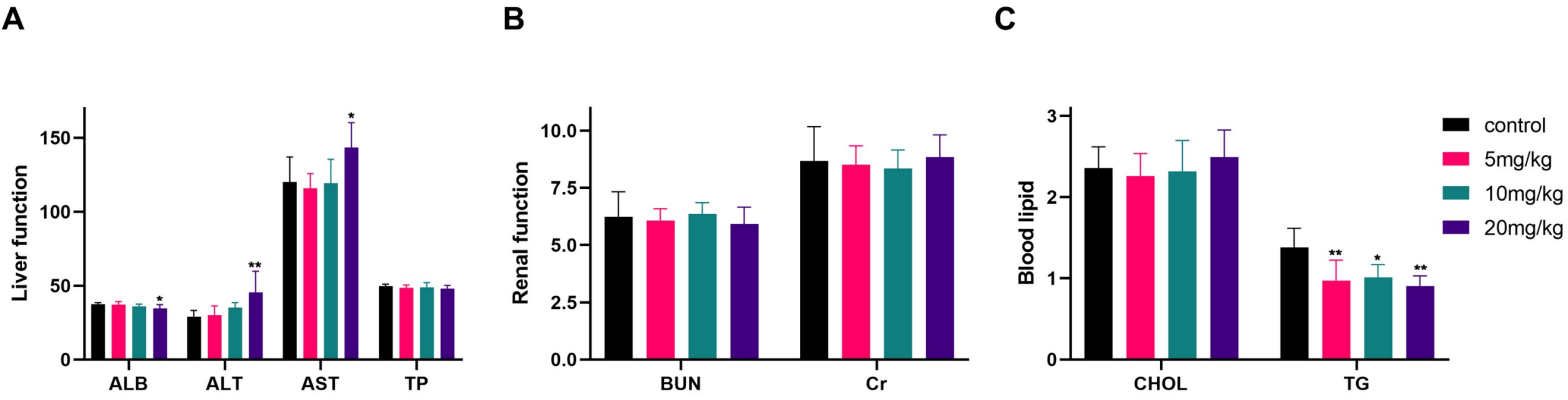
Liver, renal function and blood lipid indexes after different doses of gracillin treatment. Compared with the control group, *P < 0.05, **P < 0.01.

At the end of the experiment, serum biochemistry testing of nude mice (Fig. 14A-C) indicated significant differences between the high-dose group (20 mg/kg) and the control group in terms of ALB, ALT, AST, and TG levels, while no statistically significant differences were observed in the remaining indicators, including TP, BUN, Cr, and CHOL.

## 4. Discussion

Building on our group's previous studies, gracillin has been demonstrated to induce G1-phase cell cycle arrest in A549 lung cancer cells and promote apoptosis by regulating Bcl-2 family protein 28, as well as activate autophagy by inhibiting the mTOR signaling pathway 34. While these findings suggest a dual role for gracillin in inducing apoptosis and autophagy in lung cancer cells, its exact mechanism of action against lung cancer remains incompletely understood, necessitating further in-depth studies. In this experiment, we first verified the inhibitory effect of gracillin on the proliferation of NCI-H1299 cells through *in vitro* experiments.

Initially, we investigated the proliferation inhibition rate of gracillin on NCI-H1299 cells using the CCK-8 assay. We observed a significant inhibitory effect of gracillin on these cells, with inhibition rates increasing gradually with concentration. Programmed cell death (PCD) mechanisms, including apoptosis, necroptosis, and autophagy, play vital roles in cellular processes. Autophagy, also known as type II programmed death, is a cellular pathway involved in protein and organelle degradation, closely linked to human disease and physiology, and implicated in tumor pathogenesis 46. Among several types of autophagy, macroautophagy is the most predominant and widely studied 47, 48. Basal levels of autophagy are necessary for maintaining cellular homeostasis, but increased autophagosome formation may lead to cell death, suggesting autophagy induction as a potential strategy for promoting tumor cell death 37.

During autophagy activation, LC3-I is regulated by the phosphatase ATG4 and binds to phosphatidylethanolamine (PE) to form the LC3-II complex, crucial for autophagosome membrane genesis and maturation, and considered an autophagy hallmark 49. Beclin1, the first autophagy-associated protein identified in mammals, homologous to Atg6, forms a core complex for autophagy initiation by binding to VPS34 and VPS15^50^. P62, an extensively studied autophagic substrate, acts as a link between LC3 and polyubiquitinated proteins, selectively encapsulated into autophagosomes and eventually degraded by protein hydrolases in autophagic lysosomes 51. In this study, we conducted an in-depth investigation of autophagy-related molecular markers Beclin1, P62, and LC3 at both protein and gene levels. LC3 exhibited varying degrees of upregulation, particularly at the protein level, with the LC3-II to LC3-I ratio increasing with treatment concentration. Beclin1 showed a similar upregulation trend to LC3, while P62, although enhanced at the gene level, exhibited the opposite trend at the protein level, possibly due to its rapid degradation rate.

To observe cellular autophagy occurrence, recruitment, and aggregation of LC3 protein to autophagosomes, we employed fluorescence microscopy, confirming gracillin's ability to induce LC3 protein expression and aggregation during autophagy. Transmission electron microscopy (TEM), the gold standard for detecting autophagosomes 52, revealed a significant increase in autophagosome formation in gracillin-treated H1299 cells compared to the control group, further validating gracillin-induced autophagy. To determine whether gracillin increased LC3-II levels by blocking autophagosome-lysosome binding or by inducing autophagy, we employed chloroquine (CQ), a drug that inhibits lysosome and lysosomal binding. We found that LC3-II protein expression in the gracillin and CQ group was higher than that in the gracillin alone and CQ alone groups 53, 54. In summary, gracillin indeed induced autophagy in NCI-H1299 cells rather than inhibiting autophagosome-lysosome binding. Further studies found that pretreatment with 3-MA reversed the proliferation inhibitory effect of gracillin, suggesting that gracillin may exert its antitumor effects partly through autophagy.

Based on the transcriptome sequencing results, we propose that one potential mechanism of the anticancer effects of gracillin may involve the regulation of the mitogen-activated protein kinase (MAPK) signaling pathway, which plays a crucial role in the proliferation, differentiation, and growth of eukaryotic cells 55, 56. The MAPK signaling pathway consists of extracellular signal-regulated kinase (ERK), c-Jun-N-terminal kinase (JNK), and P38^57^. To verify whether the MAPK signaling pathway is involved in the anticancer effects of gracillin and to further elucidate its mechanism of action, we quantified the phosphorylation levels of JNK, ERK, and P38 in NCI-H1299 cells using Western blotting. The normalized results revealed that gracillin significantly upregulated the phosphorylation levels of ERK and P38 while decreasing the phosphorylation level of JNK. These observations were consistent with the changes in the MAPK signaling pathway revealed by transcriptome sequencing analysis, validating the reliability of the transcriptomics results and providing molecular-level evidence that gracillin regulates the biological behavior of lung cancer cells through the MAPK signaling pathway.

To thoroughly investigate whether gracillin-induced autophagy is dependent on the activation of p-ERK, p-P38, or the inhibition of p-JNK, we pre-treated the cells with ERK, JNK, and P38-specific inhibitors U0126, SP600125, and SB203580. The ERK signaling pathway is one of the most important signaling pathways regulating autophagy, and its activation state has been shown to induce autophagic death in lung cancer PC9 cells 58. Additionally, a study found that tretinoin was able to up-regulate LC3-II and p-ERK/ERK protein expression levels in A549 cells 59. In this experiment, when NCI-H1299 cells were pretreated with U0126 and then co-administered with gracillin, the accumulation of LC3-II was significantly inhibited, suggesting that ERK activation may play a crucial role in gracillin-induced autophagy.

JNK can regulate various cellular functions, including apoptosis and autophagy, and its activation has been shown to be essential for autophagy induction 60. However, in some cases, JNK inactivation also induces autophagy 61, 62. In this study, gracillin exhibited inhibitory effects on JNK regulation, and when cells were pretreated with JNK inhibitors, the level of LC3-II expression was higher than when cells were pretreated with gracillin alone, suggesting that further inhibition of JNK may promote autophagy. P38 is an important signaling pathway regulating cellular autophagy and apoptosis 63, 64. We observed that gracillin was able to activate the P38 signaling pathway, but surprisingly, the level of LC3-II was upregulated with the use of a P38-specific inhibitor. This finding suggests that P38 activation may not be directly related to autophagy activation but may instead promote apoptosis or other forms of cell death. Autophagy and apoptosis, as two significant cell death pathways, are intricately related to each other. It has been reported that the apoptosis inhibitor Z-VAD-FMK inhibited ginsenoside Rh2-induced apoptosis in HeLa cells, while at the same time, it increased the expression level of LC3-II in these cells 65. Additionally, activation of P38 was shown to promote the apoptotic process; for example, curcumin induced apoptosis in human lung cancer cells through activation of P38 MAPK, whereas knockdown of P38 MAPK significantly reduced curcumin-induced apoptosis 66. Based on these findings, we hypothesize that the activation of P38 here may be inducing apoptosis, and the phenomenon that inhibition of P38 increased the level of autophagy needs to be explored in future studies. In summary, gracillin-induced autophagy may be related to ERK activation and inhibition of the JNK pathway.

Based on previous studies and the analysis of high-throughput sequencing results, we observed significant expression differences in the WIPI1 gene in cells treated with gracillin. The WIPI1 gene and its encoded protein are newly discovered cellular autophagy markers involved in autophagosome formation and autophagosome-lysosome fusion. It has been suggested that the WIPI1 gene may serve as a key biomarker for autophagosome formation in certain cells 67. To elucidate the role of WIPI1 in gracillin-induced autophagy, RT-qPCR and Western blot experiments showed significant up-regulation of WIPI1, consistent with our previous findings 34. This suggests that WIPI1 may play a crucial role in gracillin-induced autophagosome formation.

Given that previous experiments have demonstrated gracillin's ability to promote autophagy by activating the ERK signaling pathway, we further explored the potential link between WIPI1 and the ERK pathway. Pretreatment of cells with the ERK-specific inhibitor U0126 led to a significant down-regulation of WIPI1 expression, comparable to that of the untreated control. This result suggests that WIPI1 may act as an effector molecule downstream of the ERK signaling pathway. Future studies will involve observing gracillin-induced autophagosome changes by overexpression and knockdown of the WIPI1 gene to clarify its role.

Furthermore, we constructed a nude mouse subcutaneous graft tumor model to explore the *in vivo* tumor suppression mechanism of gracillin. Gracillin exhibited a significant inhibitory effect on the growth of subcutaneous transplanted tumors *in vivo*, with expression levels of autophagy proteins Beclin1, LC3, P62, and WIPI1 generally consistent with the results of *in vitro* experiments. Ki-67 immunohistochemical analysis showed a significant decrease in Ki-67 protein expression in gracillin-treated tumor tissues, confirming the anti-proliferative effect of gracillin. Throughout the experiment, nude mice showed normal body weight, mental state, and appetite, with no mortality observed.

Moreover, gracillin did not induce significant toxic reactions *in vivo*, as evidenced by HE staining results of the heart, lungs, kidneys, and liver, as well as serological tests for liver and kidney function and lipid indexes. These results suggest that gracillin may be a safe and effective antitumor drug.

Although gracillin demonstrated promising *ex vivo* and *in vivo* antitumor activity, its specific mechanism of action requires further in-depth study. Future studies will focus on elucidating how gracillin exerts its antitumor effects by regulating key biological processes such as autophagy and cell proliferation, as well as its interactions with other signaling pathways. This will provide a more robust scientific foundation for the development and clinical application of gracillin as a novel antitumor drug.

## 5. Conclusion

In summary, our results suggest that gracillin possesses potent anticancer properties both *in vivo* and *in vitro*, with relatively low biotoxicity. Gracillin induces autophagy and ultimately leads to cell death, likely by activating the ERK pathway while inhibiting the JNK signaling pathway. Additionally, our findings indicate that the WIPI1 protein may act as a downstream factor in this process. With further research, gracillin holds promise as a safe and effective new treatment option for lung cancer.

## Figures and Tables

**Table 1:** The list of gene primer sequences

Gene Name	Sequence
LC3	Forward:5'-ACTGCTGCTGGGTGCTAGGAG-3'
	Reverse:5'-GTCCAAGTCCGAAGCCACAGAAC-3'
Beclin1	Forward:5'- TTGGAGGTGAGGGTGGTGATGAG-3'
	Reverse:5'-CGCCTGGGCTGTGGTAAGTAATG-3'
P62	Forward:5'-GCGTCTTGGAAAGGGTAGTGGAAG-3'
	Reverse:5'-CCTGCTGCTGGAGTTCACATCTG-3'
WIPI1	Forward:5'-CAGTAACACCGAGACGGTAC-3'
	Reverse:5'-GCACTTGGTCTGGCTACGGT-3'
GAPDH	Forward:5'-ACGGATTTGGTCGTATTGGG-3'
	Reverse:5'-TGATTTTGGAGGGATCTCGC-3'
